# Vital Statistics

**Published:** 1889-06-08

**Authors:** 


### VITAL STATISTICS.

Although this book* is modestly called "The Elements of Vital Statistics," it is something more than elementary, for it runs to 325 pages, divided into nineteen chapters. The first chapter is devoted to population; the second to the registration of births and deaths; and the third to registration of sickness. Then we have the statistics of marriages; mortality at different ages; effects of density of population on mortality; also of occupation. The mortality from zymotic diseases is fully treated of, followed by a chapter on the death-rate from special maladies. There are also life tables, and a chapter on the duration of life. Chapter 18 is devoted to "Army and Navy Statistics," and the terminal chapter is on statistical fallacies. It is satisfactory to find the author devoting a section to the latter subject, for it sometimes happens that statisticians decline perceiving there can be any fallacy where figures are concerned. But statistics are really merely a convenient aid to investigation, and not a sole method of enquiry. They constitute one of the aids of science rather than being a science in themselves. It may be admitted that the statistical method is essentially a mathematical procedure, and therefore must, as far as it goes, be correct. But it is only an attempt to give a quantitative expression to certain facts, which attempt does not always succeed, for large statistics and small statistics do not always lead to similar conclusions. Many politicians, social reformers, and amateur handlers of statistics are in the habit of drawing the conclusions that seem good to them. In fact, it has been said that figures may be so manipulated as to prove anything. Goethe said, "If figures do not govern the world, at all events they show us how it is governed." But to secure this they must be manipulated free from bias. Indeed, it has been asserted that to reason on facts of figures, and to draw conclusions from figures, is not the province of the statistician, but the business of statesmen, of political economists, and of scientific men generally.

From Dr. Newsholme's book may be culled many interesting conclusions. For example, in 1801, the population of England and Wales was 8,892,536 and of London 958,863. In 1881 the population had increased to 25,968,286 for England and Wales, and 3,814,571 for London. The registration of causes of death has given an immense impulse to sanitary work, but there are certain fallacies connected therewith, not the least being the lack of uniformity of nomenclature among medical men. With regard to the registration of disease, Dr. Newsholme observes that epidemic diarrhœa is not included in the schedule of notifiable diseases, and he thinks there are strong reasons why this should be added to the list. With this remark we fully agree, for if epidemic diarrhœa is not cholera, it is sometimes so similar to cholera that, if cholera prevailed, it would be unhesitatingly diagnosed as cholera. Especially is this the case with children—so much so that epidemic diarrhœa in children is also known as *cholera infantum.* The effect of sanitary work on the general death-rate is described at some length, and the result may be briefly stated as a reduction of mortality of 4·77 per 1,000. This implies a considerable diminution of sickness, and must be due also to a certain extent to improved methods of medical treatment, which, however, the author does not mention. It is noticeable that the mortality of children has not been reduced in the same proportion as that of adults, and Dr. Newsholme remarks, "These statistics undoubtedly appear to give countenance to the view that the strain of education produces in a certain proportion of children injurious effects on the nervous system." We have long been of opinion that "over-pressure" in education of the children of the poor—who are often taught when they ought to be satisfying hunger—is very detrimental to the health of the class.

Holding in recollection the present agitation on the subject of vaccination, we turn to Dr. Newsholme's observations on smallpox with some interest. A table is given, showing the mean annual deaths from smallpox at successive life periods, and it is evident from this table that with the gradual extension of vaccination, there has been a notable decline in the death-rate from smallpox. From another table it is seen that while the mortality from smallpox among the unvaccinated attains 64·2 per cent., the mortality of the vaccinated who afterwards have smallpox range from 8·7 to 12· per cent. only. To understand thoroughly what has resulted from vaccination, the accounts of smallpox as it prevailed a hundred years ago should be studied. Then, it was a horrible and universal disease, attacking and disfiguring nearly all—a condition of things which anti-vaccinationists, not having witnessed, do not realise.

Passing from the doleful subjects of disease and death to the more genial one of marriage, we find the author quoting Dr. Farr, who has described the marriage ratio as "the barometer of prosperity, just as the funds are the barometer of credit." This may be correct as regards the upper ranks of society, but we very much doubt whether prosperity greatly influences the marriage-rate in the lower grades of life, wherein prudential considerations do not much prevail. A table is given showing that in this country there has been a gradual decline in the number of marriages since 1874. Perhaps the recent correspondence in the *Daily Telegraph*, "Is Marriage a Failure?" may tend to render Adonis less cautious. We do not suppose the decline in the marriage-rate is attributable to any unwillingness on the part of the fairer sex, especially as there is stated to be 2,594,395 unmarried women and widows in England.

Space will not admit of a reference to a tithe of the interesting subjects discussed in this book, which ought to be on the table of every politician, social reformer, sanitarian, and medical man.

* "The Elements of Vital Statistics." By Arthur Newsholme, M.D., London; University Scholar in Medicine; Certificate in Public Health, University, London; Fellow of the Royal Statistical Society; Medical Officer of Health for Brighton. London: Swan, Sonnenschein, and Co.

